# Activity-Based Physical Rehabilitation with Adjuvant Testosterone to Promote Neuromuscular Recovery after Spinal Cord Injury

**DOI:** 10.3390/ijms19061701

**Published:** 2018-06-07

**Authors:** Dana M. Otzel, Jimmy Lee, Fan Ye, Stephen E. Borst, Joshua F. Yarrow

**Affiliations:** 1 Brain Rehabilitation Research Center, Malcom Randall Veterans Affairs Medical Center, North Florida/South Georgia Veterans Health System, Gainesville, FL 32608, USA; dana.otzel@va.gov; 2 Research Service, Malcom Randall Veterans Affairs Medical Center, North Florida/South Georgia Veterans Health System, Gainesville, FL 32608, USA; jimmylee@ufl.edu (J.L.); 2005evan@gmail.com (F.Y.); 3 Department of Applied Physiology, Kinesiology and University of Florida College of Health and Human Performance, Gainesville, FL 32603, USA; seborst@ufl.edu; 4 Division of Endocrinology, Diabetes and Metabolism, University of Florida College of Medicine, Gainesville, FL 32610, USA

**Keywords:** neuroplasticity, bodyweight-supported treadmill training, estradiol, estrogen, muscle, PI3K, IGF-1, motor neuron, BDNF, FOXO, PGC-1 alpha, PGC-1 beta

## Abstract

Neuromuscular impairment and reduced musculoskeletal integrity are hallmarks of spinal cord injury (SCI) that hinder locomotor recovery. These impairments are precipitated by the neurological insult and resulting disuse, which has stimulated interest in activity-based physical rehabilitation therapies (ABTs) that promote neuromuscular plasticity after SCI. However, ABT efficacy declines as SCI severity increases. Additionally, many men with SCI exhibit low testosterone, which may exacerbate neuromusculoskeletal impairment. Incorporating testosterone adjuvant to ABTs may improve musculoskeletal recovery and neuroplasticity because androgens attenuate muscle loss and the slow-to-fast muscle fiber-type transition after SCI, in a manner independent from mechanical strain, and promote motoneuron survival. These neuromusculoskeletal benefits are promising, although testosterone alone produces only limited functional improvement in rodent SCI models. In this review, we discuss the (1) molecular deficits underlying muscle loss after SCI; (2) independent influences of testosterone and locomotor training on neuromuscular function and musculoskeletal integrity post-SCI; (3) hormonal and molecular mechanisms underlying the therapeutic efficacy of these strategies; and (4) evidence supporting a multimodal strategy involving ABT with adjuvant testosterone, as a potential means to promote more comprehensive neuromusculoskeletal recovery than either strategy alone.

## 1. Introduction

Spinal cord injury (SCI) results in profound sensorimotor impairment below the lesion site, which is precipitated by the insult to the spinal cord and exacerbated by a cascade of secondary cellular and molecular processes occurring in the acute and chronic post-injury phases [1,2,3]. Neuromuscular impairment and muscle loss are hallmarks of the SCI injury cascade that produce substantial impediments to physical therapy regimens intended to restore locomotion [3]. Activity-based physical rehabilitation therapies (ABTs) (e.g., bodyweight-supported treadmill training (BWSTT) or robotic assisted locomotor training with or without electric stimulation) have shown promise in promoting neuromuscular plasticity in humans and rodent models after motor-incomplete SCI [4,5]. However, ABTs are only partially effective in restoring function after SCI and their neuromuscular efficacy declines as injury severity increases [6], indicating the need for adjuvant therapeutic strategies to hasten functional recovery in this population.

Low testosterone (T) is also a secondary consequence of SCI, with hypogonadism being ~four times more prevalent in men after SCI than in non-injured populations [7]. Non-neurologically impaired men with low T exhibit low muscle mass, impaired muscle function [8], and worsened walking biomechanics [9], while T replacement therapy (TRT) improves muscle mass, neuromuscular function [10], and walking speed in older ambulatory hypogonadal men [11]. To-date, a causal relationship between low T and worsened neuromuscular function after SCI has not been elucidated, although TRT increases sublesional lean mass [12] and muscle cross-sectional area (CSA) in men with motor-complete SCI [13], in a manner completely independent from voluntary muscle contractility. Similarly, in male and female rodent SCI models, androgen treatment attenuates sublesional muscle loss and other phenotypic changes associated with impaired muscle function after SCI [14,15,16,17,18,19,20], and may promote slight improvement in locomotor recovery [15]. Herein, we will summarize the (1) molecular deficits underlying muscle loss after SCI; (2) independent influences of T and ABTs on neuromuscular function and musculoskeletal integrity subsequent to SCI; (3) hormonal and molecular mechanisms underlying the therapeutic efficacy of these strategies; and (4) evidence supporting a multimodal strategy involving BWSTT with adjuvant T, as a potential means to promote more comprehensive neuromusculoskeletal recovery after SCI.

## 2. Neuromuscular Adaptations after SCI

Motoneuron innervation of skeletal muscle fibers influences muscle mass and function, and fiber-type distribution [21]. After SCI, changes to the excitatory and inhibitory inputs to the motor unit (i.e., spinal motoneuron and the innervated fibers) exist [22]. For example, motor units that are dysfunctional after SCI result in a weaker and less fatigue resistant muscle [22,23], with rate of force development and motor axon conduction velocity decreasing [24,25]. These changes may exacerbate functional decline after SCI and can impair locomotor recovery, depending on the injury severity and degree of motor dysfunction [26]. The plethora of anatomical and functional consequences resulting from motor unit impairment after SCI have been detailed elsewhere [22]. Herein, we will focus on somatic motoneuron structural adaptations that influence impaired motor function after SCI [27] and how T administration [17,18] and locomotor training [28,29] may alter these adaptations and ultimately improve muscle function and/or locomotor recovery.

The muscular adaptations to SCI have been detailed previously [30], which we summarize here in brevity. Individuals with SCI exhibit an 18–46% lower sublesional muscle cross-sectional area (CSA) than those without SCI, with the degree of atrophy depending on the injury severity [31] and muscle groups involved [32,33,34]. Muscle loss is worsened as injury severity progresses [31], a result of more impaired motor function and extended disuse in those with motor-complete SCI. For example, muscle atrophy occurs primarily in the initial six weeks after injury in those with motor-incomplete SCI [32], while whole muscle CSA and muscle fiber (f)CSA continually decline for at least 24 weeks after motor-complete SCI [33,35]. Atrophy of both type I (slow-oxidative) and type II (fast-glycolytic) fibers occurs in humans [35,36] and rodents in response to SCI [37], preceding intramuscular fat accumulation [32] and the well-characterized slow-to-fast fiber transition [38,39]. These structural and physiologic changes impair voluntary force generation and muscle endurance [40,41], factors that are associated with locomotor recovery after SCI [42,43]. However, skeletal muscle retains the ability to reverse the molecular cascade precipitating atrophy [44,45] and to improve contractility after SCI in response to sufficient stimuli [46]. In the sections below, we discuss evidence supporting the involvement of several molecular signaling pathways that likely influence muscle deficits after SCI.

### 2.1. Ubiquitin-Proteasome Signaling after SCI

Reduced phosphorylation of the forkhead box O (FOXO) proteins FOXO1 and FOXO3a promotes the transcription of muscle atrophy F-box (MAFbx or atrogin-1) and muscle ring finger-1 (MuRF1), muscle-specific E_3_ ubiquitin ligases that stimulate atrophy in response to disuse and other catabolic states [47]. The influence of these ubiquitin ligases on skeletal muscle atrophy has been demonstrated by the viral overexpression of MAFbx, which reduced the myotube diameter by ~85% in vitro, and by genetic elimination of MAFbx or MuRF1, which attenuated muscle atrophy in response to sciatic nerve transection [48]. Within several days of SCI, >50 protein ubiquination pathway genes are upregulated in sublesional muscle [49], an effect that likely influences the rapid muscle atrophy in response to injury. In particular, MAFbx and MuRF1 mRNA expressions are increased five- to >40-fold within two to eight days of injury [50,51,52,53,54], with protein expressions elevated >two-fold prior to the initiation of muscle atrophy [55]. Interestingly, the fold inductions of MAFbx and MuRF1 were associated with the rate of skeletal muscle atrophy in response to spinal cord transection [53], suggesting that a higher expression of these genes exacerbates muscle loss after SCI. In this regard, FOXO1 mRNA was 33% higher [54] and MAFbx and MuRF1 mRNA were >two-fold higher in rodents after spinal transection than in animals receiving sciatic nerve transection [53], potentially explaining the more rapid atrophy occurring after SCI than in other disuse conditions [53,56]. However, the increased expressions of these and other ubiquitin proteasome pathway genes do not persist after experimental SCI [49], with FOXO1, MAFbx, and MuRF1 gene expressions and pFOXO1 and pFOXO3 proteins reverting to the level of uninjured controls within two to 10 weeks of spinal cord transection [53,54,57]. Similarly, in humans with motor-complete SCI, MAFbx and MuRF1 expressions are high at one-month post-injury and decline by 50–75% at three- and 12-months, resulting from reductions in pFOXO1 and/or total FOXO3 proteins [58]. Indeed, in cohorts of men with chronic SCI, MAFbx and MuRF1 gene expression appear equal to [59] or less than uninjured controls, with FOXO1, FOXO3a, and MAFbx proteins being >35% lower than controls [60]. While our discussion focused on changes in the ubiquitin-proteasome pathway, it is important to note that other catabolic pathways likely influence muscle atrophy after SCI. For example, calpain 1 mRNA expression is increased as early as day 4 after spinal cord transection, an effect that persists for at least 15 days [50], which is important given the influence of calpain signaling on myofibrillar proteolysis [61].

### 2.2. IGF-1 Signaling Pathway after SCI

IGF-1 mediated activation of the phosphatidylinositol-3 kinase (PI3K)/Akt signaling pathway promotes skeletal muscle anabolism [62]. In the immediate days after SCI, mRNA encoding IGF-1, the IGF-1 receptor (IGF-1R), and several IGF binding proteins (IGFBP) are upregulated in muscle [50,51,63], and muscle IGF-1 protein expression is increased [63]. Despite this, muscle pAkt is reduced within several days of SCI [51,55], while relatively normal total and phosphorylated 4E-BP1 and S6K1 expression persist in the first few weeks after SCI [50,51]. The inability of IGF-1 to increase pAkt and downstream targets acutely after SCI likely occurs because insulin receptor substrate-1 (IRS1) protein expression is rapidly reduced in response to SCI [51]. In this regard, IRS1 expression is required for IGF-1 mediated muscle hypertrophy, evidenced by the inability of genetic IGF-1 overexpression to promote skeletal muscle hypertrophy in IRS1 deficient animals [64]. Subsequently, the downregulation of IGF-1 signaling persists chronically after SCI, evidenced by (1) 25–40% lower circulating IGF-1 [65,66] and 50% lower IGF-1 protein expression in the muscle of individuals with chronic SCI compared with controls [60]; and (2) lower total Akt protein and lower total and phosphorylated mTOR and S6K1 in rodent muscle 10-weeks after spinal cord transection [57,67], accompanying 50% lower immunoprecipitated S6K1 activity [67]. Similarly, in humans with motor-complete SCI, total and phosphorylated mTOR and regulatory-associated protein of mTOR (RPTOR), an mTOR cofactor, were lower at three- and 12-months after injury, when compared with values obtained one-month post-SCI, likely influencing the lower p4E-BP1 and pS6 (subunit of S6K1) protein expression [58] that persists for upwards of 30 years after SCI [60]. In this regard, circulating IGF-1 has been shown to be highly correlated with increased thigh muscle CSA in men with chronic motor-complete SCI [68].

### 2.3. PGC-1α and PGC-1β Signaling after SCI

In skeletal muscle, peroxisome proliferator-activated receptor gamma co-activator (PGC)-1α regulates type I fiber expression and mitochondrial biogenesis [69], while PGC-1β regulates various aspects of mitochondrial structure and function [70]. As evidence, transgenic PGC-1α expression results in a whole body muscular phenotype that is high in type I oxidative fibers [71] and muscle-specific PGC-1α overexpression increases mitochondrial biogenesis [72]. In comparison, ablation of PGC-1β in skeletal muscle myofibers in adulthood reduced mitochondrial CSA and mitochondrial respiration without altering fiber-type distribution, resulting in increased oxidative stress and impaired exercise performance [70]. Baligand et al. used genome wide analysis and identified that genes associated with mitochondrial dysfunction and impaired oxidative phosphorylation were altered three to 14 days after SCI [49]. In particular, muscle PGC-1α mRNA expression was suppressed to a nearly undetectable level three days after spinal cord transection [54] and within two weeks, muscle PGC-1α protein was dramatically reduced, accompanying reductions in slow myosin heavy chain (MHC) protein expression [73] that is indicative of fewer oxidative fibers. Similarly, at eight-weeks post-transection, muscle PGC-1α gene expression and total and nuclear PGC-1α protein were >50% lower than controls, and slow MHC and slow troponin mRNA and protein were >60% lower [16]. However, at 17-weeks after spinal cord transection, muscle PGC-1α mRNA expression was no longer suppressed in rodents [74]. In this regard, the hallmark slow-to-fast fiber-type shift resulting from spinal cord transection is most pronounced in the first 100 days after injury, with relatively minor changes in type I MHC expression thereafter [38]. Regardless, muscle PGC-1α mRNA was 75% lower in men with chronic motor-complete SCI than in normally active men [75]. In comparison, relatively less is known about the changes in muscle PGC-1β after SCI. Interestingly, PGC-1β mRNA was reduced by 90% within three days of sciatic nerve transection [54], an effect that is mentioned because SCI typically results in relatively larger gene expression changes [76] and more rapid muscle loss than other disuse models [53,56]. Wu et al. also reported that muscle PGC-1β mRNA expression was 50% lower than controls at eight-weeks after spinal transection [74] and Kramer et al. reported 75% lower PGC-1β mRNA in men with chronic motor-complete SCI, when compared with ambulatory controls [75].

## 3. Androgenic Regulation of Muscle

**Figure 1:**
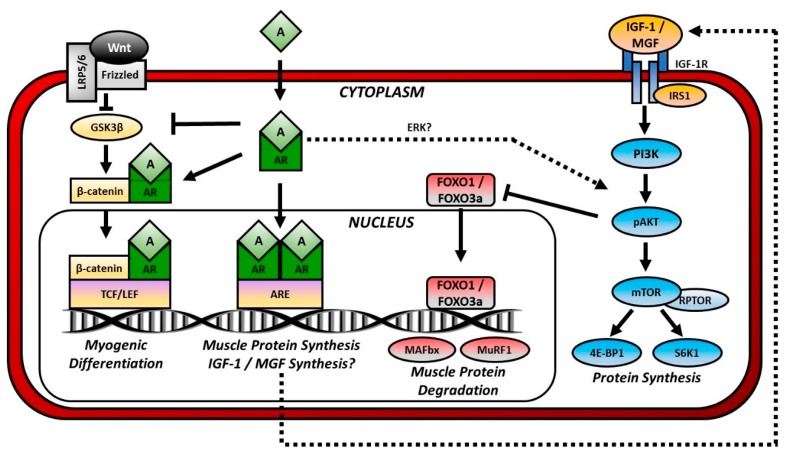
Androgen-mediated Anabolic and Anticatabolic Signaling Pathways in Muscle. Anabolic signaling: Androgens (A) pass through the plasma membrane and bind to cytosolic androgen receptors (AR). Dimerized and phosphorylated ARs pass through the nuclear membrane and bind to a region of the DNA termed the androgen response element (ARE), thereby initiating protein synthesis. Ligand-bound ARs may also enhance Wnt signaling as follows. Wnt binds to Frizzled and in turn disheveled (not shown). Disheveled inhibits the activity of glycogen synthase kinase-3β (GSK3β), which phosphorylates β-catenin and marks it for degradation. When GSK3β is inhibited, β-catenin accumulates and enters the nucleus where it binds to a region of the DNA termed the T-cell factor/lymphoid enhancer factor (TCF/LEF) that regulates genes involved in myogenic differentiation. Ligand-bound ARs enhance Wnt signaling by inhibiting GSK3β and attaching to β-catenin for nuclear shuttling. Androgens may also indirectly stimulate protein synthesis by activating the phosphatidylinositol-3 kinase (PI3K)/Akt signaling through actions of Erk or by promoting synthesis of insulin-like growth factor (IGF)-1 or mechano growth factor (MGF). IGF-1 and MGF bind cell-surface IGF-1 receptors (IGF-1R) and activate PI3K/Akt signaling. Anticatabolic signaling: Activation of PI3K/Akt signaling inhibits the transcription factor forkhead box O (FOXO). FOXO1 and FOXO3a activate muscle atrophy F-box (MAFbx or atrogin-1) and muscle ring finger-1 (MuRF1), and E_3_ ubiquitin ligases that prepare proteins for proteasome degradation.

Maintenance of skeletal muscle mass requires relative balance between muscle protein synthesis (i.e., anabolic processes) and protein degradation (i.e., catabolic processes), with elevated catabolic signaling and/or reduced anabolic signaling influencing muscle loss. Readers are directed to the following in-depth review of the various signaling pathways that influence skeletal muscle anabolism and catabolism in response to disuse [77]. Herein, we will discuss how androgens influence muscle mass via classical AR-mediated signaling and through interactions with other intercellular anabolic and catabolic signaling pathways (Figure 1).

### 3.1. Testosterone Synthesis and Metabolism

Testosterone is the most abundant bioactive androgen and is primarily synthesized by testicular Leydig cells in males [78] and by the ovaries in females [79], and to a lesser extent in other organs, such as the adrenal cortex [80]. The majority of T circulates are bound to protein transporters (i.e., sex-hormone binding globulin (SHBG) or albumin), with only a small fraction (~1–2%) circulating freely unbound to any protein transporter [81]. The fraction of free (i.e., unbound) T and albumin-bound T is bioavailable [82] because it can bind cell surface or cytosolic androgen receptors (ARs), in the case of free T, or cross the cell membrane and dissociate from its low affinity protein transporter, in the case of albumin, which allows subsequent cytosolic AR binding. In comparison, SHBG-bound T is biologically inactive because it cannot (1) bind cell-surface/plasma-membrane ARs; (2) cross cellular membranes and bind cytosolic ARs [83]; or (3) undergo enzymatic metabolism [84]. As such, elevated SHBG is associated with reduced androgenic action in target tissues expressing ARs, including skeletal muscle [78], bone [84], and spinal motoneurons [85,86,87], among others.

In addition to serving as a hormone, T is a substrate for the tissue-specific synthesis of two bioactive sex-steroid hormones, dihydrotestosterone (DHT) and estradiol (E_2_), via actions of the 5α-reductase and aromatase enzymes, respectively [78,84]. The localized conversion of T to DHT, via any of the three 5α-reductase isozymes [78], enhances tissue-specific androgen signaling given that DHT binds to ARs with ~three times the affinity of T [88] and that DHT maintains a longer presence in tissues because it is not aromatized to E_2_ nor converted to androstenedione (a weaker androgen), via 17β-hydroxysteroid dehydrogenase (17β-HSD) [78]. Similarly, the aromatization of T to E_2_ amplifies estrogen signaling in tissues expressing estrogen receptors (ERs) [89]. In males, this conversion primarily occurs in non-gonadal tissue, with >80% of circulating E_2_ derived from localized peripheral metabolism [90]. However, in females, the peripheral aromatization of T to E_2_ also amplifies estrogen signaling [79], an effect that is particularly evident after the menopausal transition or in other situations where ovarian estrogen synthesis declines [80].

### 3.2. Hypogonadism and Testosterone Replacement after SCI

After SCI, 45–60% of men exhibit low T (hypogonadism, T < 300 ng/dL) [91,92,93] that results, in-part, from secondary testicular dysfunction [94] and impairments in the hypothalamic-pituitary axis [95,96]. Hypogonadism is ~four times more prevalent after SCI than in able-bodied men [7], with T concentrations remaining >25% below that of age-matched healthy men for an extended duration after SCI [91,97]. Additionally, a 50% greater decline in circulating T and >10-fold higher increase in SHBG occurs throughout the age-span in response to SCI [92], indicating that assessment of total T may underestimate hypogonadism incidence after SCI, a concept that we have demonstrated in other populations that exhibit elevated SHBG [81]. A causal relationship between low T and muscle loss after SCI has yet to be identified, although it is interesting to note that Finkelstein et al. reported that healthy men receiving pharmacologic treatment to maintain circulating T in the subphysiologic range (191 ± 78 ng/dL) exhibited a significant decline in whole body lean mass and thigh muscle area [98]. In this regard, the median T concentrations observed in several SCI cohort studies (i.e., 160–220 ng/dL) [93,99] were similar to that of Finkelstein et al., suggesting that the magnitude of hypogonadism after SCI is sufficient to induce muscle loss.

Our rodent severe contusion SCI model also exhibits 40–55% lower circulating T than controls for at least two months after injury [14,15,37,100] and androgen treatment attenuates muscle loss [14,15,16] and fCSA atrophy [17,18,19,20] in some, but not all, sublesional muscles in rodent SCI models [16,37,101]. In this regard, androgen-induced muscle preservation appears dependent upon muscle-specific AR expression, given that Phillips et al. reported muscle preservation in the sublesional levator ani/bulbocavernosus (LABC) muscle (high AR expression), but not soleus (low AR expression), in response to T treatment after SCI [37]. Importantly, neither E_2_ treatment [17] nor the conversion of T to DHT [14] influence muscle preservation in rodent SCI models, suggesting dispensability of aromatase and 5α-reductase activity in muscle. Indeed, Borst et al. [102] demonstrated that co-administration of T with finasteride (type II 5α-reductase inhibitor) to non-neurologically impaired men resulted in dramatically lower DHT, but did not prevent T-induced improvements in lean mass or muscle strength. Several small clinical trials have also assessed TRT in men with motor-complete SCI. For example, Cooper et al. observed ~40% lower urinary protein and creatinine excretion in SCI patients receiving TRT acutely after injury [103,104], indicating reduced whole body and muscle protein catabolism. Additionally, Bauman et al. reported that 12 months of TRT increased upper- and lower-extremity lean mass and resting energy expenditure in hypogonadal men with motor-complete SCI [12], with improvement persisting for at least 12-months after TRT discontinuation [105]. Lastly, a single case-study recently reported that TRT increased thigh muscle CSA in a man with motor-complete SCI [13].

### 3.3. Classical Androgen Signaling

Classical androgen-mediated genomic signaling is initiated by the binding of T or DHT to cytosolic ARs to form hormone-receptor complexes that translocate to the nucleus and bind androgen response elements (AREs) [106], resulting in the activation or repression of multiple gene pathways that regulate skeletal muscle [107]. Similarly, E_2_ stimulates classical estrogen-mediated genomic signaling that is characterized by binding cytoplasmic ERα or ERβ, which stimulates nuclear translocation of the hormone-receptor complex and binding to estrogen response elements (EREs). ER signaling is pronounced in bone [89], although the aromatization of T to E_2_ is not obligatory for skeletal muscle maintenance [98].

Androgens also exert actions that are independent of nuclear AREs [78]. For example, androgens [78] and estrogens [108] exert effects by binding non-traditional cell-surface sex-steroid hormone receptors (e.g., G-protein-coupled receptors (GPCR)) that rapidly alter several intracellular signaling pathways [106,109]. In mesenchymal multipotent C3H 10T1/2 cells, ligand-bound ARs also complex with cytosolic β-catenin [110]. This interaction results in the chaperoning and nuclear accumulation of the androgen-AR/β-catenin complex and stimulation of downstream T-cell factor (TCF)-4 genes, an effect that simultaneously promotes myogenic differentiation and suppresses adipogenic differentiation of mesenchymal pluripotent cells [110]. For further discussion on Wnt/β-catenin signaling, readers are directed to the following in-depth review [111]. Similarly, in C2C12 myotubes [112] and rodent skeletal muscle [113], androgens increase the phosphorylation of cytosolic glycogen synthase kinase-3β (GSK3β), which (1) inhibits the phosphorylation, ubiquitination, and degradation of β-catenin; and (2) promotes nuclear β-catenin accumulation and TCF activity. Ligand-bound ARs also interact with cytosolic β-catenin in motoneurons [114] and neuronal gonadotropin-releasing hormone cell lines [115] to promote nuclear shuttling of β-catenin and TCF-4 binding, which influences β-catenin-dependent transcriptional activity. To our knowledge, the functional significance of androgen-AR/β-catenin nuclear shuttling in motoneurons has not been determined. However, the loss of endogenous ARs accelerates motoneuron degeneration [116] and pharmacologically-induced stimulation of β-catenin protein expression in the spinal cord is associated with improved locomotor function after SCI [117,118,119], suggesting that nuclear β-catenin accumulation may promote motoneuron function.

### 3.4. Androgenic Crosstalk with IGF-1 and PI3K/Akt Signaling

Ligand-bound ARs activate PI3K/Akt signaling in muscle indirectly by stimulating the expression of (1) IGF-1 [113,120] and/or (2) other intracellular signaling molecules (e.g., Erk) that may activate or suppress components of this pathway independently of IGF-1 [121]. In support of this contention, the upstream promoter of the human *IGF-1* gene exhibits two AREs that activate IGF-1 expression [122]. In rodents, androgen administration increases IGF-1 and mechano growth factor (MGF) mRNA expression by several fold in skeletal muscle within seven days of administration [113], an effect that persists for at least one month [120]. Similarly, within one month of initiating TRT, older men exhibit a >two-fold increase in IGF-1 protein expression in muscle [123]. In cultured rodent myoblasts, T treatment stimulates protein accretion and mammalian target of rapamycin (mTOR) mRNA expression, effects that are abrogated by co-incubation with bicalutamide (competitive AR antagonist), rapamycin (mTOR inhibitor), or LY294002 (PI3K inhibitor) [121,124], indicating that T-induced protein accretion is dependent upon both AR activation and PI3K signaling. Similarly, dose-dependent phosphorylation of Akt, mTOR, S6 (subunit of S6K1), and S6K1 occurs in cultured rodent and C2C12 myotubes in response to increasing concentrations of T [125,126]. Importantly, these effects are also abolished by co-incubation with LY294002, Akt inhibitor VIII, or rapamycin [126], demonstrating dependence upon the PI3K signaling pathway. Further, complete androgen withdrawal was sufficient to reduce muscle IGF-1 mRNA and suppress Akt, mTOR, S6K1, and 4E-BP1 phosphorylation (downstream PI3K targets) by >50% in rodents, with androgen (nandrolone or DHT) treatment increasing these factors two-fold above gonadally-intact animals [125,127]. Interestingly, Serra et al. reported that orchiectomy also increased RPTOR and reduced tuberous sclerosis complex protein 2 (TSC2) phosphorylation by >50%, changes associated with mTOR inhibition, while T treatment reversed these effects [128]. These findings indicate that androgens stimulate various IGF-1/PI3K/Akt signaling components in muscle and suggest that activation PI3K/Akt signaling influences androgen-mediated muscle hypertrophy. This contention is strengthened by the observation that MKR mice, which express a dominant negative IGF-1R in mature muscle fibers, exhibit attenuated hypertrophy of the highly androgen-sensitive LABC muscle in response to T treatment [128]. Regardless, IGF-1 and IGF-1R mRNA expression was not altered in animals receiving nandrolone for eight weeks after spinal cord transection, suggesting that androgens do not chronically stimulate IGF-1 expression in muscle in the absence of descending supraspinal input and/or mechanical strain [16].

### 3.5. Androgen Crosstalk with the Ubiquitin-Proteasome Pathway

Androgenic influence on FOXO expression and subsequent downstream signaling is evidenced by (1) increased total FOXO1 and FOXO3a protein and lower pFOXO3a in rodent muscle in response to orchiectomy (ORX) [125,128]; (2) 10- to 25-fold higher MAFbx and MuRF-1 mRNA expression in rodent skeletal muscle within seven days of ORX [128,129], an effect that persists for at least one month, albeit to a lesser magnitude over time [120,125]; and (3) the ability of androgen treatment to abolish these changes and prevent ORX-induced muscle atrophy [120,125,128,129]. Similarly, TRT suppresses ubiquitin-proteasome activity in the muscle of older men [130]. In C2C12 myoblasts and myotubes, androgen-induced suppression of MAFbx occurs indirectly via interactions among ligand-bound ARs and octamer binding transcription factor (Oct)-1 or Ankryn repeat domain protein 2 (Ankrd2), co-regulators of sex-steroid hormone induced transcriptional activity in target genes [131,132]. Interestingly, androgen treatment also attenuated muscle atrophy and the increases in FOXO1, MAFbx, and MuRF1 mRNA expression resulting from methylprednisolone administration after SCI [101], an effect that may be mediated by androgenic suppression of glucocorticoid receptor expression [120,133,134]. Alternatively, it is also possible that androgens indirectly influence FOXO signaling by stimulating PI3K/Akt signaling in muscle, given that FOXO1 and FOXO3a are downstream targets of pAkt signaling [62], although we are unaware of any study addressing this interesting possibility.

### 3.6. Androgenic Influence on PGC-1α and PGC-1β Signaling after SCI

Testosterone stimulates muscle PGC-1α expression via direct AR-mediated and/or ER-mediated pathways following aromatization to E_2_. As evidence, (1) castration lowered muscle PGC-1α mRNA by 50% in male rodents and nandrolone treatment increased PGC-1α mRNA >two-fold [125]; (2) ovariectomy lowered muscle PGC-1α >70% in female rodents [135], while one week of E_2_ replacement increased PGC-1α mRNA and nuclear protein expression in postmenopausal women [136]; and (3) within eight days of initiating pharmacologic E_2_ therapy, muscle PGC-1α mRNA was increased by 30% in men [137]. As discussed above, PGC-1α [16,54] and -1β expressions [74] are suppressed in response to spinal cord transection, an effect that may influence the slow-to-fast fiber transition [30] and/or mitochondrial dysfunction after SCI [138]. In this regard, chronic androgen treatment increased PGC-1α mRNA >three-fold in the muscle of animals receiving spinal cord transection and prevented the SCI-induced reductions in total and nuclear PGC-1α protein, changes that were accompanied by higher slow troponin mRNA and protein expression and lower fast troponin mRNA [16]. Similarly, Gregory et al. reported that androgen treatment attenuated the slow-to-fast fiber transition after spinal cord transection [20]. In addition, Wu et al. observed >two-fold higher PGC-1β mRNA expression in the muscle of spinal cord transected animals receiving nandrolone for eight weeks, although this change did not reach the level of statistical significance [16]. Regardless, the role of aromatase in mediating the influence of androgens on muscle PGC-1α and/or PGC-1β expression remains to be determined after SCI.

## 4. Androgenic and Estrogenic Regulation of Motoneurons

The influence of SCI on somatic motoneuron structure depends largely upon injury characteristics and the muscle groups innervated (discussed in [27]). For example, Sengelaub et al. [17] and Byers et al. [18] reported no differences in quadriceps motoneuron counts or soma area four-weeks after mid-thoracic contusion SCI, although, dendritic length was decreased 40–50%. Similarly, four-weeks after mid-thoracic spinal transection, fewer dendrites (labeled via sciatic nerve) were present on spinal motoneuron, and dendritic arbor size and dendritic length were reduced in comparison to intact animals [28]. Reduced tibialis anterior and soleus motoneuron dendritic length was also observed four-months after mid-thoracic contusion SCI, with no difference in soma area [29]. Whereas, Bose et al. reported fewer motoneurons innervating the soleus at four-months after mid-thoracic contusion SCI, with residual motoneurons being relatively larger and having fewer total dendrites that exhibited longer dendritic lengths [27]. When taken together, these findings indicate that dendrite length is rapidly reduced after SCI, with the loss of relatively small motoneurons and short dendrites occurring thereafter.

ARs are expressed in the cytoplasm and nuclear regions of spinal motoneurons [86] in a roughly equal distribution among male and female rodents [85]. 5α-reductase type II is highly expressed in several gray- and white-matter areas of the spinal cord [139], including spinal motoneurons [140]. Spinal cord homogenates and cultured NSC34 cells (immortalized motoneurons) actively convert T to DHT [141] in a manner that is ~three-times greater than that occurring in other central nervous system (CNS) regions [142] or the seminal vesicles, an androgen-sensitive tissue that highly expresses 5α-reductase type II [143]. The neuronotrophic effects of androgens are evidenced by the ability of T and DHT to equally preserve motoneuron number and size in cultured rodent lumbosacral spinal cord segments [144]. Indeed, lumbosacral motoneurons innervating the quadriceps and other musculature are androgen responsive [145]. However, the androgen sensitivity of spinal motoneurons appears largely dependent upon the magnitude of AR expression in motoneurons and in the innervated skeletal muscle, given that (1) motoneurons innervating LABC (high AR expression) exhibit robust dendritic atrophy in response to ORX, which is completely prevented by T or DHT administration [146]; (2) motoneurons innervating quadriceps (relatively lower AR expression) do not exhibit dendritic atrophy in response to ORX [147]; and (3) transgenic AR overexpression in skeletal muscle increased somatic motoneuron androgenic responsiveness, evidenced by ORX-induced dendritic atrophy in normally non-androgen responsive motoneurons, which was completely prevented by T administration [147].

ERα and ERβ are also expressed in cytoplasmic and nuclear regions of spinal motoneurons [148,149] and cultured motoneurons actively synthesize E_2_ in culture [149], owing to aromatase expression [148,149]. However, cultured rodent embryonic and adult spinal cord homogenates do not appear to synthesize E_2_ when incubated with androstenedione [142] or T [141]. Regardless, E_2_-mediated preservation of motoneuron dendritic growth [150] is as robust as that resulting from T or DHT treatment [151] and is highly dependent upon motoneuronal ERα expression, at least during development [152]. In culture, E_2_ treatment also improves spinal motoneuron cell survival during glutamate-induced toxicity, an effect that was not inhibited by ICI 182,780 (competitive ERα/ERβ agonist) [153], indicating that estrogen-mediated improvement in spinal motoneuron viability occurs independent of nuclear ER-signaling. In this regard, GPCR-30 (or G protein-coupled estrogen receptor-30) has been identified as a cell-surface/transmembrane ER that rapidly stimulates intracellular signaling [108] and E_2_ has been shown to improve motoneuron viability during glucose-oxygen deprivation, an effect that was blocked by co-treatment with G15 (GPCR-30 inhibitor) [154].

### 4.1. Influence of Androgens and Estrogens on Motoneuron Structure after SCI

As discussed above, altered dendritic length is a hallmark of SCI that has been observed in motoneurons innervating sublesional vastus lateralis [17,18], tibialis anterior [29], and soleus muscle [27]. In adult female rats, T treatment completely prevented the reduction in dendritic length of quadricep motoneurons resulting from SCI, without altering motoneuron counts, soma volume, or other motoneuron structural variables [18]. Whereas, DHT or E_2_ treatment each attenuated the reduction in quadriceps dendritic length by 50–60% [17]. The functional consequences of preserved dendritic length after SCI remains to be determined. However, it is interesting to note that our laboratory has reported that T treatment slightly increased hindlimb locomotor recovery in male rodents subsequent to moderate-severe contusion SCI, with the largest benefit occurring in response to supraphysiologic T [15], while E_2_ treatment is known to produce comparatively larger locomotor improvements in both male and female rodents after SCI [155].

### 4.2. Molecular Pathways Underlying Androgen-Mediated Motoneuron Protection

Androgenic regulation of brain-derived neurotrophic factor (BDNF) and tyrosine receptor kinase (Trk)B (BDNF receptor) in spinal motoneurons appears particularly important for motoneuron anatomical preservation [156,157]. As evidence, both somatic motoneurons and the spinal nucleus of the bulbocavernosus (SNB) express BDNF and TrkB mRNA and protein [146,158], with >80% of motoneuron dendrites identified as TrkB-positive [159]. In male rodents, ORX reduced BDNF mRNA and protein expressions in SNB [159] and in motoneurons innervating the quadriceps [158], while T treatment prevented these effects. BDNF regulates soma size independently of T, as evidenced by the ability of exogenous BDNF to maintain SNB motoneuron soma size in ORX male rats after bilateral axotomy [160]. In comparison, both BDNF and T appear necessary for the maintenance of SNB dendritic length, given that neither exogenous BDNF nor T independently prevented the reduction in SNB motoneuron dendritic length resulting from bilateral axotomy in ORX males, while co-treatment with BDNF and T increased dendritic length [161]. In females, T treatment also increased SNB motoneuron count by 50%, an effect that was prevented by the co-administration of TrkB-IgG [162]. These findings are particularly striking and suggest that androgen treatment may represent a means of stimulating BDNF/TrkB signaling, given the prevalence and severity of hypogonadism after SCI [91,92,93] and that the stimulation of motoneuron BDNF/TrkB signaling enhanced functional recovery in rodent SCI models [163].

In addition, estrogenic activation of GPCR-30 [108] or ERα [164] stimulates intercellular PI3K/Akt signaling, which promotes motoneuron survival [155]. As evidence, treatment of cultured spinal motoneurons with LY294002 (PI3K inhibitor) blocked the anti-apoptotic effects of E_2_ treatment during glucose-oxygen deprivation, resulting in a 50% increase in apoptotic motoneurons [154]. Similarly, PC12 cell viability was reduced ~50% upon treatment with rapamycin (mTOR inhibitor), demonstrating the necessity of downstream PI3K/Akt/mTOR signaling for cell survival [165]. Interestingly, LY294002 co-administration suppressed E_2_-mediated voluntary locomotor recovery in male rodents after SCI [154], indicating that PI3K/Akt signaling influences locomotor recovery after SCI. Estrogens may also exert neuroprotection via other pathways that have been reviewed by others [155].

## 5. Activity-Based Physical Rehabilitation after SCI

After SCI, increasing focus has been placed on improving activity-dependent neuromuscular plasticity through the use of BWSTT, a therapy that involves robotic or manual placement of the impaired limbs into normal gait patterns on a slowly moving treadmill, or other ABTs (e.g., passive or functional electric stimulation cycling) [166]. Through intense repetitive practice, it is postulated that ABTs activate sublesional spinal networks that promote beneficial neuromuscular adaptations by retraining the CNS to recover task-specific motor activities, via stimulation of the central pattern generator (CPG) in the lumbosacral region of the spinal cord [166]. Indeed, a number of studies involving persons with motor-incomplete SCI indicate that BWSTT produces several functional benefits, including (1) improved temporal gait parameters associated with walking ability (e.g., increased number of steps, faster cadence, and improved muscle activation patterns) [167,168,169,170,171,172,173,174,175,176,177]; (2) improved muscle strength and rate of torque development in impaired limbs, and reduced detrimental co-activation of antagonist muscle groups [24,169,171,178,179,180]; and (3) reduced muscle atrophy [24,178,181,182,183,184] (Table 1). However, when data from well-controlled randomized clinical trials assessing BWSTT after SCI [179,185,186,187,188] are pooled, there appears to be only limited meaningful improvement in overground walking speed or distance [4,189,190,191], given that the minimal clinically important difference in walking speed is reported as 0.13 m/s in the SCI population [192]. This finding stresses the necessity of testing combinatory therapies that prime the neuromuscular system for better functional recovery when combined with BWSTT.

Preclinical studies also utilize ABTs to evaluate mechanisms underlying activity-dependent neuroplasticity after SCI and have observed locomotor and neuromuscular improvements in response to BWSTT (Table 2) [5]. However, there are considerable differences in the reported effects of BWSTT on locomotor and neuromuscular recovery in SCI rodents, in-part due to the influence of age and gender, the mechanism of producing SCI (i.e., transection, hemisection, or contusion), delay in initiating BWSTT, duration of therapy, and the methods of assessing neuromuscular recovery [5,193]. In general, better outcomes for locomotor recovery are observed in female rats with mild to moderate contusion SCI, with training that begins seven to 14 days after injury and continues for at least eight weeks [5]. In this regard, female rodents have higher endogenous E_2_ than males [194], which is important given that subphysiologic and physiologic E_2_ treatment significantly improves open-field locomotor recovery in both male and female rodents after SCI, in-part, by reducing secondary apoptotic and inflammatory damage to the spinal cord [155].

### 5.1. Effects of ABTs on Muscle

In rodent SCI models, BWSTT produces relatively consistent improvements in muscle integrity, even following complete transection. As evidence, after spinal transection, the incorporation of BWSTT completely preserved the muscle mass:body mass ratio [195,196] and prevented ~55% of soleus fCSA loss [196]. Similarly, in a moderate-contusion SCI model, BWSTT produced 23% higher soleus fCSA and 38% higher soleus peak tetanic force in comparison with untrained SCI animals [197], with analogous effects reported by others [49,197,198,199,200]. Similarly, several case-report and cohort-studies have reported muscle responses in individuals with motor-incomplete SCI. For example, in persons with chronic SCI, four to six months of BWSTT increased vastus lateralis muscle fCSA [178,184]. Likewise, Jayaraman et al. reported that nine-weeks of BWSTT enhanced knee extensor and ankle plantar flexion voluntary muscle activation and increased plantar flexion peak torque by 43%, which accompanied a 15% increase in plantar flexor CSA [201], an important finding given that lower extremity strength is positively associated with walking function after SCI [42]. However, others have reported much less robust strength improvements [202,203] or no strength improvement [171] in response to BWSTT.

The structural improvements mentioned above appear to be influenced, in-part, by reduced catabolic signaling resulting from BWSTT. As evidence, in a rodent SCI model, three sessions of BWSTT suppressed >10 protein ubiquitination pathway genes, an effect accompanied by the full preservation of muscle mass [49]. Similarly, after spinal cord transection, five sessions of electrical stimulation of paralyzed muscle suppressed MAFbx and MuRF1 mRNA and myostatin mRNA in muscle [51]. Myostatin is a member of the transforming growth factor (TGF)-β family and a muscle-derived negative regulator of muscle growth, which acts via the activin IIB receptors [204]. Additionally, muscle regeneration occurs in response to BWSTT after SCI, evidenced by the upregulation of myogenic regulatory factors and increased expression of Pax7 positive nuclei (a marker of satellite cell activation) [63,205], suggesting that BWSTT stimulates muscle anabolic signaling pathways. In this regard, IGF-1, MGF, IGF-1R, and several IGFBPs mRNA were increased 1.5 to >10-fold in rodent muscle and IGF-1 protein expression was increased >three-fold within seven days of initiating BWSTT after moderate-contusion SCI [63]. In contrast, other reports indicate that electrical stimulation of paralyzed muscle suppressed IGFBP-4 and -5 mRNA in muscle, without altering IGF-1 or MGF mRNA after spinal transection [51,206]. Similarly, no change in circulating IGF-1 was observed in persons with chronic SCI undergoing a comprehensive physical rehabilitation program involving one to two hours per week of bodyweight-supported locomotor training [207]. As such, the role of IGF-1 signaling in the muscle regenerative response to BWSTT requires further elucidation, especially given that SCI-induced downregulation of IRS1 limits IGF-1 mediated activation of PI3K/Akt signaling in muscle acutely after SCI [51]. 

In addition to muscle preservation, BWSTT is reported to reduce soleus fatigue and attenuate the slow-to-fast fiber transition occurring in rodent moderate-contusion SCI models [197]. Similarly, in persons with chronic SCI, four to six months of BWSTT partially reversed the slow-to-fast fiber transition, evidenced by increased oxidative fibers and fewer type IIx (glycolytic) fibers compared with the baseline [178,184]. Interestingly, lower *Mhyl*, *Mybph*, and *Myh4* gene expression (fast-twitch fiber markers) was apparent in rodent muscle within five-days of initiating BWSTT [49], indicating that reversal of the slow-to-fast fiber-type switch occurs rapidly with this physical rehabilitation modality. However, to our knowledge, changes in PGC-1α or -1β have not been evaluated in response to BWSTT after SCI. Regardless, a five-fold increase in PGC-1α mRNA in muscle was observed in men with chronic motor-complete SCI within several hours of neuromuscular electrical stimulation of quadriceps [208] and a ~two-fold increase in PGC-1α was present in men with chronic motor-complete SCI undergoing functional electric stimulation for durations of 16-weeks [209] to six-years [210], suggesting that increased PGC-1α expression may underlie the slow-to-fast fiber-type reversal occurring in response to BWSTT.

### 5.2. Effects of ABTs on Motoneurons

In rodent SCI models, ABTs limit injury severity, evidenced by the preservation of myelin, axons, and collagen morphology and decreased lesion volume [198], and produce a host of anatomical and functional improvements to the motor unit [29,198,200,211,212,213]. For example, after spinal cord transection, BWSTT increases axonal regrowth [212], preserves motoneuron dendritic length, increases neuromuscular junction synaptic density in the lumbar motoneuron pool [29], and produces larger group Ia afferent-evoked monosynaptic excitatory postsynaptic potentials (EPSPs) in the ankle plantar flexors, an effect that is particularly prevalent in rats regaining the greatest stepping ability [213]. Similarly, after moderate-severe contusion SCI, BWSTT produced a beneficial reduction in the sprouting of small caliber afferent fibers and improved stepping patterns while on the treadmill [200], although open-field locomotor activity did not improve in this study, suggesting some limitation to functional recovery in more severe injury scenarios. Interestingly, Singh et al. induced a spinal transection at nine weeks following contusion SCI in a subgroup of rats that previously underwent BWSTT or no training and observed that kinematic improvements were maintained in BWSTT rats and worsened in untrained rats, indicating that functional improvement resulting from BWSTT occurred via neural networks originating below the lesion level [200]. Indeed, Cote el al. reported that after spinal transection, BWSTT facilitated lumbar spine motoneuron pool recruitment via improved reflex pathways resulting from afferent input [211].

In individuals with incomplete SCI, beneficial adaptations to spinal neuronal pathways have been demonstrated in response to BWSTT. For example, the soleus H-reflex phase-dependent modulation during walking normalizes homosynaptic facilitation, reverses homosynaptic depression, and improves the presynaptic inhibition of Ia afferents [214,215,216]. However, better functional recovery has also been associated with increased ankle dorsiflexor and knee extensor maximal motor-evoked potential, a probe of corticospinal tract excitability, in persons with motor-incomplete SCI undergoing BWSTT [180] and improved ankle dorsiflexor and plantar flexor muscle co-activation patterns accompany improved walking function in response to BWSTT [171], findings that indicate greater descending corticospinal drive. In this regard, improvements in the above-mentioned factors likely stem from the reorganization of both supraspinal and spinal cord neural circuits in response to BWSTT [214,217], with further research needed to resolve whether functional adaptations rely more heavily on improved cortical or spinal circuitry in individuals with SCI.

While the molecular signals regulating BWSTT-induced motoneuron recovery after SCI require further elucidation, there is growing evidence that several inducible neurotrophic factors influence neuroplasticity [211]. For example, an upregulation of BDNF and TrkB (BDNF receptor) mRNA and protein has been observed in the skeletal muscle and spinal cord of healthy animals in response to voluntary wheel running or forced treadmill running, with BDNF protein levels increasing to a greater degree in the spinal cord than in the soleus [237,256,257]. In this regard, BDNF and TrkB both undergo retrograde transport from skeletal muscle to the spinal cord [258], an effect that may mediate locomotor recovery after SCI by improving synaptic transmission and plasticity, axon regeneration, and motoneuron survival [259]. Indeed, in rodent SCI models, BWSTT upregulates BDNF mRNA and protein in skeletal muscle and the spinal cord [29,196,198,211,237,260,261,262,263,264,265,266,267] and TrkB protein in the lumbar spinal cord [237,257]. Interestingly, Leech and Hornby also observed increased circulating BDNF in persons with motor-incomplete SCI undergoing treadmill training, with high-intensity treadmill exercise (performed at 100% peak gait speed) resulting in the larger BDNF increases than exercise performed at 33% or 66% peak gait speed [268]. In addition, BSWTT upregulated glial cell derived neurotrophic factor (GDNF) protein in the lumbar spinal cord, which was positively associated with the facilitation of motoneuron excitability [211].

Neurotrophin-3 (NT-3) is another endogenous neurotrophin that regulates synaptic transmission, promotes neuromuscular junction maturation, and improves the survival and function of sensory neurons via the activation of TrkC (receptor), both of which are expressed in the spinal cord [269,270,271,272,273]. Indeed, recent results from Fang et al. indicate that co-treatment with BDNF/TrkB and NT-3/TrkC promoted motor recovery in a rodent SCI model [274]. NT-3 is produced in the spinal cord and NT-3 mRNA and protein levels are upregulated after seven days of voluntary treadmill and wheel running in healthy rodents [256,262,265]. Similarly, following SCI, rodents exhibit increased NT-3 mRNA and protein in the spinal cord within seven days of initiating BWSTT [262,263,265,266]. Interestingly, Ying et al. reported that BWSTT did not increase spinal cord NT-3 protein expression in adult male rats after spinal cord hemisection [266], while Cote et al. observed increased NT-3 protein levels in the lumbar and thoracic spinal cord after four weeks of BWSTT in adult female rats with complete transection [211], suggesting that sex differences may exist in the NT-3 response to BWSTT. In this regard, E_2_ treatment increases NT-3 levels in several CNS regions [275], although we are unaware of any study evaluating whether spinal cord NT-3 expression is regulated via E_2_-mediated mechanisms. BWSTT is also reported to upregulate genes involved in neuroplasticity (e.g., *Arc* and *Nrcam*) and angiogenesis (e.g., *Adam8* and *Tie1*), which may be important given that improved neurovascular remodeling has the potential to improve locomotor function after SCI [235].

## 6. Testosterone Adjuvant to BWSTT

We are unaware of published results from any randomized study evaluating T treatment as an adjuvant to BWSTT. However, at least one ongoing study is evaluating TRT as an adjuvant to electric stimulation-based resistance training in men with motor-complete SCI [276]. A single case-report has also indicated that a man with motor-complete SCI exhibited increased thigh muscle CSA in response to twice-weekly neuromuscular electric stimulation-based resistance training, when combined with adjuvant TRT [13]. In addition, our preliminary data indicates that the combination of testosterone-enanthate (TE), a long-acting T ester [277], and quadrupedal BWSTT (consisting of two 20-min bouts performed twice daily, five-days per week for seven-weeks) promoted more complete neuromuscular restoration than TE-alone in our rodent moderate-severe contusion SCI model [278]. Specifically, the incorporation of TE+BWSTT after SCI produced near-complete restoration of the sublesional muscle mass:body mass ratio, completely prevented the slow-to-fast fiber-type transition occurring in the soleus, attenuated the reduction in soleus fCSA by >50%, and maintained isolated soleus muscle force mechanics near the level of intact control animals. Impressively, 100% of SCI animals receiving TE+BWSTT regained the ability to perform occasional voluntary overground weight-supported hindlimb plantar stepping within seven-weeks of initiating therapy, while 0/11 SCI animals and only 2/10 of SCI animals receiving TE-alone regained this ability. As such, our preliminary data appear to support the contention that a combinatory strategy involving BWSTT with adjuvant TE promotes musculoskeletal and neuromuscular improvement in male rodents after moderate-severe contusion SCI.

### Potential Side-Effects of TRT

TRT is approved for the treatment of hypogonadism in adult men and has been shown to increase muscle protein synthesis in women [279]. However, given its androgenic potential, chronic TRT remains controversial for women, at least when administered in the doses necessary to produce musculoskeletal benefit in males. This should not detract from TRT research in relation to SCI because men represent ~80% of new SCI cases [280] and many of these men will develop hypogonadism of an indefinite duration [91,92,93]. Regardless, careful evaluation of TRT safety and efficacy is necessary prior to implementation of this therapy after SCI.

To-date, meta-analyses have confirmed that TRT produces three adverse events (AEs) in non-neurologically impaired adult men: (1) polycythemia, which occurs in ~6% of men treated with TRT; (2) a higher number of cumulative prostate-related events (discussed below); and (3) a slight decrease in HDL cholesterol, which is of unknown clinical significance [281]. The development of polycythemia in men receiving TRT has raised concerns regarding the cardiovascular safety of TRT, especially when taken in context with findings from the Testosterone in Older Men with Mobility Limitations (TOM) trial [282] and from two retrospective studies that indicated increase cardiovascular risk in hypogonadal men receiving TRT [283,284]. In this regard, the TOM trial was discontinued upon recommendation of the Data Safety Monitoring Board due to a higher prevalence of cardiovascular-related AEs in the TRT versus placebo groups [282]. However, this trial received criticism because of the relatively poor classification of cardiovascular AEs [285]. In comparison, the NIH-funded T-trials, which represent the largest randomized clinical trials to-date evaluating the safety and efficacy of TRT, reported no differences in adjudicated cardiovascular AEs in hypogonadal men receiving TRT versus placebo [11]. Similarly, heavy scientific criticism and numerous calls for retraction [286] accompanied the retrospective studies from Vigen et al. [284] and Finkel et al. [283] that reported increased cardiovascular risk in hypogonadal men receiving TRT. Indeed, the aforementioned findings remain in direct conflict with findings from the largest meta-analyses on the topic, which reported that TRT does not increase cardiovascular risk in men enrolled in well-controlled clinical trials [287,288], and with findings from the largest retrospective study (*n* = 83,010 men with low T) that indicated TRT reduced all-cause mortality by 56% and MI and stroke risk by 24–36% in comparison with untreated hypogonadal men [289]. Of note, the potential cardiovascular responses to TRT have not been extensively evaluated in the SCI population. However, in a small clinical trial, La Fountaine et al. reported that QTa interval variability was higher in hypogonadal men with SCI than in eugonadal men with SCI, and that TRT normalized QTa interval variability, suggestive of reduced arrhythmia risk [290].

The risk for increased cumulative prostate-related events also merits mention because prostate growth and worsened urinary symptoms resulting from TRT have the potential to worsen bladder dysfunction, which is already common in the SCI population [291]. In this regard, the prostate-related AEs associated with TRT appear to be driven primarily by the 5α-reduction of T to DHT [8]. As evidence, we have reported that older hypogonadal men receiving TE-alone exhibited a 43% increase in prostate volume over a period of 12-months, while men receiving TE plus finasteride exhibited no change in prostate volume [102]. In comparison, 5α-reductase inhibition did not prevent the musculoskeletal or lipolytic benefits resulting from TRT in our trial [102] or others [292,293], indicating that finasteride co-treatment improves the prostate-related safety profile of TRT without inhibiting musculoskeletal efficacy in older men. The safety and efficacy of TE plus finasteride treatment in men with SCI currently remains unknown, although an ongoing clinical trial in our laboratory is evaluating this promising combinatory therapy in hypogonadal men with SCI [294]. In addition, it is important to note that a 15-yr retrospective study of 150,000 men reported that TRT was not associated with prostate cancer [295] and that no meta-analysis to-date has reported increased prostate cancer risk resulting from TRT [8].

Several less common risks are also associated with TRT, which we have previously reviewed [296], including: pain or bleeding at injection sites, skin reactions, fluid retention, breast tenderness, gynecomastia, and liver disorders. Importantly, these side-effects remain putative [8] and have not been confirmed by meta-analysis [281]. However, one potential risk that merits mention is the possibility that TRT may cause or worsen obstructive sleep apnea [297], a condition that is relatively common in tetraplegic men with SCI [298]. As such, need exists to determine TRT safety in men with SCI who display obstructive sleep apnea, especially given the higher sleep apnea prevalence in non-neurologically impaired men receiving TRT [297].

## 7. Conclusions

Upregulation of FOXO signaling occurs in the immediate days to weeks after SCI, an effect that likely influences the rapid muscle loss in this population. Similarly, muscle PGC-1α and -1β are reduced in response to SCI, underlying the slow-to-fast fiber change and impaired mitochondrial function subsequent to SCI. In comparison, downregulation of IGF-1 and PI3K/Akt signaling occurs more chronically after SCI. These molecular changes are precipitated by the spinal injury and subsequent disuse, but may also be exacerbated by low T that occurs in many men with SCI. T promotes muscle anabolism via direct AR-mediated genomic signaling and/or via cross-talk with IGF-1 and the PI3k/Akt signaling pathway. T may also promote anticatabolic effects in muscle by directly or indirectly suppressing FOXO signaling. Further, androgen treatment increases PGC-1α in muscle and attenuates the slow-to-fast muscle fiber-type transition occurring in response to spinal transection. In addition, T improves the preservation of motoneuron dendritic length after SCI directly via AR-mediated mechanisms and/or indirectly, following aromatization to E_2_. Regardless, these improvements appear to result in only minor improvement in locomotor recovery in rodents after moderate-severe contusion SCI, likely because only limited descending corticospinal input remains. In comparison, BWSTT improves descending corticospinal drive and/or facilitates reorganization of spinal cord neural circuitry below the lesion, which produces neuromuscular improvement and some restoration of musculoskeletal integrity. However, findings from well-controlled randomized clinical trials indicate that BWSTT only promotes limited clinically meaningful improvement in voluntary locomotor recovery in humans after SCI. Interestingly, activity-mediated neuromuscular plasticity appears to rely, in-part, upon the upregulation of BDNF/TrkB in the spinal cord. Androgen/AR signaling is required for BDNF/TrkB-mediated dendritic restoration in androgen sensitive motoneurons in the lumbosacral spinal cord, suggesting that T treatment may enhance activity-mediated neuromuscular plasticity after SCI. Indeed, our preliminary data supports this contention, given that adult male rodents receiving TE adjuvant to BWSTT exhibited profound musculoskeletal and neuromuscular improvement and recovery of voluntary weight-supported overground plantar stepping after moderate-severe contusion SCI. Elucidation of the molecular mechanisms underlying these neuromusculoskeletal and locomotor improvements will assist in validating our hypothesis and will improve the likelihood for the translation of this promising multimodal therapeutic strategy.

## Figures and Tables

**Table 1:** Characteristics of Bodyweight Supported Locomotor Training in Human Spinal Cord Injury Studies.

Study	Intervention	Duration	Population	N	Outcomes
**Studies Assessing Gait**					
Dobkin et al., 2007 [185]	Manual-assisted BWSTT vs. OG training	12 wk	38 ASIA B, 107 ASIA C&D	145	BWSTT increased walking speed from ~0.40 m/s at 2 weeks post-entry to 0.85 m/s for ASIA C&D. OG training increased walking speed from ~0.50 at 2 weeks post-entry to 0.84 m/s for ASIA C&D. Poor walking outcomes were observed for ASIA B participants.
Field-Fote et al., 2001 [167]	BWSTT with ES	3 days/wk, 12 wk	ASIA C	19	Walking speed increased from 0.12 to 0.21 m/s. ^a^
Alcobendas-Maestro et al., 2012 [218]	Robotic-assisted BWSTT vs. OG training	3–6 mo	ASIA C&D	48 M/32F	Robotic-assisted BWSTT and OG training walking speed remained the same from baseline to post-training. Robotic-assisted BWSTT increased 6 min walk distance from 91 to 169 m. ^a^
Alexeeva et al., 2011 [179]	BWS training on a fixed track vs. BWSTT vs. Comprehensive PT	3 days/wk 13 wk	ASIA C&D	30 M/5 F	BWS on a fixed track increased walking speed from 0.33 to 0.44 m/s. ^a^ BWSTT increased walking speed from 0.30 to 0.46 m/s. ^a^ PT increased walking speed from 0.41 to 0.51 m/s. ^a^
Duffell et al., 2015 [202]	Robotic-assisted BWSTT	3 days/wk, 4 wk	ASIA C&D	19 M/7 F	Walking speed increased from ~0.55 to ~0.58 m/s. ^a^
Esclarin-Ruz et al., 2014 [219]	Robotic-assisted BWSTT + OG training (LKOGT) vs. Conventional OG training (OGT)	5 days/wk, 8 wk	ASIA C&D	59 M/24 F	LKOGT increased walking speed from 0.48 to 0.54 m/s and 6 min walk distance from 122 to 187 m ^b^ in participants with upper motor neuron injury. LKOGT increased walking speed from 0.24 to 0.46 m/s and 6 min walk distance from 82 to 157 m ^b^ in participants with lower motor neuron injury. OGT increased walking speed from 0.36 to 0.39 m/s and 6 min walk distance from 93 to 119 m in participants with upper motor neuron injury. OGT increased walking speed from 0.28 to 0.45 m/s and 6 min walk distance from 94 to 145 m in participants with lower motor neuron injury.
Field-Fote et al., 2005 [220]	Manual-assisted BWSTT (TM) vs. BWSTT + ES (TS) vs. BWS OG training + ES (OGS) vs. Robotic-assisted BWSTT (LR)	5 days/wk 12 wk	ASIA C&D	22 M/5 F	TM increased walking speed from ~0.07 to ~0.10. TS increased walking speed from ~0.12 to ~0.16 m/s. ^a^ OG increased walking speed from ~0.14 to ~0.19 m/s. ^a^ LR increased walking speed from ~0.09 to ~0.11.
Field-Fote & Roach 2011 [186]	Manual-assisted BWSTT (TM) vs. BWSTT + ES (TS) vs. BWS OG training +ES (OGS) vs. Robotic-assisted BWSTT (LR)	5 days/wk, 12 wk	ASIA C&D	52 M/12 F	TM increased walk speed from 0.17 to 0.22 m/s ^a^ and 2 min distance from 22.1 to 23.0 m. TS increased walk speed from 0.18 to 0.23 m/s ^a^ and 2 min distance from 20.6 to 24.4 m. ^a^ OGS increased walk speed from 0.19 to 0.28 m/s ^a^ and 2 min distance from 24.0 to 38.3 m. ^a,b^ LR increased walk speed from 0.17 to 0.18 m/s and 2 min distance from 16.8 to 17.9 m.
Gorassini et al., 2009 [169]	Manual-assisted BWSTT	5 days/wk, 14 wk	ASIA C&D	14 M/3 F	In 9 responders, walking speed increased from 0.31 to 0.55 m/s. ^a^ In 8 non-responders, there was no change in walking speed.
Harkema et al., 2012 [170]	Manual-assisted BWSTT	3 day/wk, 4 to 92 wks	ASIA C&D	148M/48 F	Walking speed increased from 0.31 to 0.51 m/s ^a^ and 6 min walk distance increased from 91 to 154 m. ^a^
Kapadia et al., 2014 [221]	Robotic-assisted BWSTT + ES	3 days/wk, 16 wk	ASIA C&D	13 M/3 F	Walking speed increased from 0.23 to 0.28 m/s and 6 min walk distance increased from 187.9 to 217.1 m. ^a^
Knikou 2013 [214]	Manual-assisted BWSTT	5 days/wk, 1.5–3.5 mo	ASIA C&D	9 M/3 F	6 min walk distance increased from 36.25 to 39.05 m for ASIA C and from 252 to 279.5 m for ASIA D participants.
Krishnan et al., 2016 [171]	Robotic-assisted BWSTT	3 day/wk, 4 wk	ASIA C&D	8 M/8 F	Median walking speed increased from 0.58 to 0.66 m/s. ^a^ 6 min walking distance did not change.
Labruyere et al., 2014 [222]	Robotic-assisted BWSTT	4 days/wk, 4 wk	ASIA C&D	5 M/4 F	Walking speed increased from 0.62 to 0.66 m/s.
Lam et al., 2015 [223]	Robotic-assisted BWSTT with resistance (LR) vs. Robotic-assisted BWSTT only (LO)	3 days/wk, 12 wk	ASIA C&D	9 M/6 F	LR increased walking speed from 0.29 to 0.40 m/s. LO increased walking speed from 0.33 to 0.44 m/s.
Lucarelli et al., 2011 [187]	BWSTT vs. Conventional gait training	2 days/wk, 12 wk	ASIA C&D	20 M/10 F	BWSTT increased walking speed from 0.85 to 1.25 m/s ^a^ as well as increased cadence, distance, step length and swing phase. Conventional training did not improve gait quality or speed.
Morrison et al., 2018 [224]	Manual-assisted BWSTT	120 sessions	ASIA C&D	49 M/20 F	Median walking speed increased by 0.25 m/s. ^a^ Median 6 min walk distance increased by 66 m. ^a^
Niu et al., 2014 [225]	Robotic-assisted BWSTT	3 days/wk, 4 wk	ASIA C&D	27 M/13 F	Walking speed increased in the low-walking capacity group from 0.12 to 0.15 m/s and in the high-walking capacity group from 0.84 to 0.97 m/s. 6 min walk distance did not change.
Nooijen et al., 2009 [172]	Manual-assisted BWSTT vs. Manual-assisted BWSTT with ES vs. OG training with ES vs. Robotic-assisted BWSTT	4 days/wk, 12 wk	ASIA C&D	40 M/11 F	All therapies led to small improvements in gait quality (increased cadence and step length) with no differences among groups.
Potsans et al., 2004 [188]	BWSTT with ES Cross-over design: treatment-control (AB); control-treatment (BA)	5 days/wk, 4 wk	ASIA C&D	12 M/2 F	In AB group, walking speed increased 0.23 m/s and 6 min walk distance increased 72.2 m. In BA group, walking speed increased 0.17 m/s and 6 min walk distance increased 63.8 m.
Thomas et al., 2005 [180]	Manual-assisted BWSTT	4 days/wk, 16 wk	ASIA C&D	8 M/2 F	Walking speed increased from 0.15 to 0.53 m/s ^a^ and 6 min walk distance increased from 34.2 to 167.6 m.
Varoqui et al., 2014 [203]	Robotic-assisted BWSTT	3 days/wk, 4 wk	ASIA C&D	14 M/ 1 F	Walking speed increased from 0.56 to 0.64 m/s ^a^ and 6 min walk distance did not change (207 to 209 m).
Winchester et al., 2005 [226]	Robotic-assisted BWSTT	3 days/wk 12 wk	ASIA C&D	4 M	Walking speed increased for 3 participants from 0.0 to 0.11, 0.0 to 0.81, 0.24 to 0.62 m/s and one remained unable to ambulate.
Wirz et al., 2005 [175]	Robotic-assisted BWSTT	4 days/wk, 8 wk	ASIA C&D	18 M/2 F	Walking speed increased from ~0.37 to ~.48 m/s ^a^ and 6 min walk distance 120 to 160 m. ^a^
Wu et al., 2012 [227]	4 wk Robotic-assistance BWSTT + 4 wk Robotic-resistance BWSTT	3 days/wk, 8 wk	ASIA D	8 M/2 F	Walking speed increased from 0.67 to 0.76 m/s ^a^ and 6 min walk distance increased from 223 to 247 m. Step length and cadence increased. ^a^
**Studies Assessing Muscle**					
Duffell et al., 2015 [202]	Robotic-assisted BWSTT	3 days/wk, 4 wk	ASIA C&D	19 M/7 F	Maximal isometric ankle dorsiflexion torque increased from 12.3 to 13.2 Nm, ^a^ but plantar flexion torque did not change (28.1 vs. 28.4 Nm).
Galen et al., 2014 [228]	Robotic-assisted BWSTT	5 days/wk, 6 wk	ASIA C&D	14 M/4 F	Percent peak torque increased 68% for hip flexion, 54% for hip extension, 93% for knee flexion and 71% for knee extension.
Gorassini et al., 2009 [169]	Manual-assisted BWSTT	5 days/wk, 14 wk	ASIA C&D	14 M/3 F	In 9 responders, peak electromyography activity increased from 67 to 135 μV in the tibialis anterior muscle and 36 to 50 μV in the hamstrings. In 8 non-responders, there was no change.
Jayaraman et al., 2008 [201]	Manual-assisted BWSTT	5 days/wk, 9 wk	ASIA C&D	4 M/1 F	Isometric knee extension strength increased 21%. Isometric plantar flexion strength increased 44%. Knee extension and plantar flexion voluntary muscle activation improved. Maximal CSA of the plantar flexors increased and 15%.
Krishnan et al., 2016 [171]	Robotic-assisted BWSTT	3 day/wk, 4 wk	ASIA C&D	8 M/8 F	BWSTT increased isometric ankle dorsiflexion by 20% and ankle plantar flexion by 22%.
Thomas et al., 2005 [180]	Manual-assisted BWSTT	4 days/wk, 16 wk	ASIA C&D	8 M/2 F	Peak electromyography activity averaged from four lower limb muscles increased during treadmill walking from 82.4 to 137.1 μV.
Varoqui et al., 2014 [203]	Robotic-assisted BWSTT	3 days/wk, 4 wk	ASIA C&D	14 M/1 F	Ankle dorsiflexion torque increased from 26.8 to 29.1 Nm^a^ and ankle plantar flexion torque increased from 10.9 to 13.5 Nm. ^a^

BWSTT = bodyweight supported treadmill training; CSA = cross-sectional area; ES = electrical stimulation; m = meter, min = minute; mo = month; OG = overground; PT = physical therapy; wk = week. Note: ^a^ indicates statistically different from baseline; ^b^ indicates statistically different between training groups.

**Table 2:** Characteristics of Bodyweight Supported Treadmill Training (BWSTT) in Rodent SCI Studies.

Study	Sex/Age	Injury Level	Start of Training	Training Duration	BBB wk 1	BBB End	Gait Outcome	Muscle/Electrophysiology
**Studies Assessing BWSTT after Spinal Hemisection**								
Maier et al., 2009 [229]	F/A	T8	1 wk	8 wk	~12	N/R	Stepping ↑	N/R
Battistuzzo et al., 2016 [230]	M/A	T10	1 wk	9 wk	N/R	N/R	Kinematics ↑	N/R
Battistuzzo et al., 2017 [231]	M/A	T10	1 wk	9 wk	N/R	N/R	N/R	G fCSA ↑
Shah et al., 2013 [232]	N/R/N/R	T10	5 days	2.5 wk	N/R	N/R	Stepping ↑	N/R
Goldshmit et al., 2008 [212]	N/R/A	T12	1 wk	4 wk	~3	11T/6C	Kinematics ↑	G-S fCSA ↑
**Studies Assessing BWSTT after Mild Contusion SCI**								
Nessler et al., 2006 [233]	F/Y	T9	1 wk	12 wk	13	14T/No C	N/R	N/R
Oh et al., 2009 [234]	M/Y	T9–10	1 wk	4wk	~4	13T/8C	N/R	N/R
**Studies Assessing BWSTT after Moderate Contusion SCI**								
Stevens et al., 2006 [197]	F/Y	T8	1 wk	1 wk	~5	10T/6C	N/R	Sol F ↑/fCSA ↑
Liu et al., 2008 [199]	F/Y	T8	8 days	12 wk	3–7	15T/11C	N/R	G-S CSA ↑
Nessler et al., 2006 [233]	F/Y	T9	1 wk	12 wk	9	11T/No C	N/R	N/R
Shin et al., 2014 [235]	F/Y	T9	1 wk	8 wk	~6	~13T/~10C	N/R	N/R
Wang et al., 2015 [29]	F/Y	T9	1 wk	16 wk	~7	~13T/~11C	N/R	N/R
Singh et al., 2011 [200]	F/Y	T9–10	1 wk	8 wk	~5	~8T/~9C	N/R	mass/bw ↑
Bose et al., 2012 [198]	F/A	T8	1 wk	12 wk	~3	~15T/~11C	Stepping ↑	N/R
Multon et al., 2003 [236]	F/A	T9	2–4 days	12 wk	~2	10T/8C	N/R	N/R
Wu et al., 2016 [237]	F/A	T10	1 wk	4 wk	~5	~13T/~9C	N/R	N/R
Foret et al., 2010 [238]	F/A	T10	1 day	4 wk	~2	~9T/~7C	N/R	N/R
Ward et al., 2014 [239]	M/Y	T8	2 wk	12 wk	~7	~12T/~9C	N/R	Sol EMG ↑
Park et al., 2010 [240]	M/Y	T10	3 days	25 days	~2	11.5	N/R	N/R
Liu et al., 2017 [241]	N/R/N/R	T9	1 wk	2 wk	N/R	N/R	Stepping ↑	N/R
**Studies Assessing BWSTT after Severe Contusion SCI**								
Hayashibe et al., 2016 [242]	F/Y	T8–9	1 wk	4 wk	<1	16T/10C	N/R	N/R
Heng et al., 2009 [243]	F/Y	T9	42 days	8 wk	N/R	N/R	Stepping ↑	N/R
Nessler et al., 2006 [233]	F/Y	T9	1 wk	12 wk	4.5	~9T/No C	N/R	N/R
Shinozaki et al., 2016 [244]	F/Y	T10	6 wk	8 wk	~1	4T/3C	N/R	N/R
Robert et al., 2010 [245]	F/A	T7–8	2 wk	2 wk	~3	4T/3.5C	N/R	N/R
Ichiyama et al., 2009 [246]	F/A	T10	30 days	8 wk	N/R	N/R	Gait not improved	N/R
**Studies Assessing BWSTT after Spinal Transection**								
Petruska et al., 2007 [213]	F/N	T6–8	16 days	6 wk	N/R	N/R	Stepping ↑	G EPSP ↑
Tillakaratne et al., 2010 [247]	F/N	T7–8	26 days	8 wk	N/R	N/R	Stepping ↑	N/R
See et al., 2013 [248]	F/N	T8–9	3 wk	4 wk	N/R	N/R	Kinematics ↑	N/R
Timoszyk et al., 2005 [249]	F/N	N/R	64 days	40 days	N/R	N/R	Gait not improved	N/R
Zhang et al., 2007 [250]	F/Y	T8	5 days	40 days	<1	~6.5T/~2C	N/R	N/R
Lee et al., 2010 [251]	F/Y	T8	3 wk	42 wk	N/R	N/R	Gait not improved	N/R
De Leon et al., 2006 [252]	F/Y	T9	3 wk	16 wk	N/R	N/R	Kinematics ↑	N/R
Moshonkina et al., 2004 [253]	F/Y	T9	1 day	9 wk	N/R	N/R	Kinematics ↑	N/R
Kubasak et al., 2008 [254]	F/N/R	T9	4 wk	20 wk	N/R	N/R	Gait not improved	N/R
Fouad et al., 2000 [255]	B/A	T8	3 days	5 wk	~9	14T/14C	Kinematics ↑	N/R
Ihla et al., 2011 [196]	M/A	T8–9	6 days	9 wk	N/R	N/R	N/R	Sol fCSA ↑

Sex: B, both females and males; F, female; M, male; N/R, not reported; Age: A, adult; N, neonate; N/R, not reported; Y, young; Injury Level: T, thoracic; Start of Training: wk, week; BBB end of training: T, trained; C, control SCI untrained; Other gait outcome: ↑ improvement; Muscle/Electrophysiology: bw, bodyweight; EPSP, evoked monosynaptic excitatory postsynaptic potentials F, force; fCSA, fiber cross-sectional area; G, gastrocnemius; G-S, gastrocnemius-soleus; ↑, increased; Sol, soleus, N/R, not reported. Note: The BBB scale ranges from 0–21 and is used to evaluate functional locomotor recovery ranging from no observable movement (0) to consistent weight-supported plantar stepping with coordinated gait (21).

## Abbreviations

ABT	activity-based therapies
AR	androgen receptor
ARE	androgen response element
BDNF	brain-derived neurotrophic factor
BWSTT	bodyweight-supported treadmill training
CNS	central nervous system
CSA	cross-sectional area
DHT	dihydrotestosterone
E_2_	estradiol
EPSP	excitatory postsynaptic potential
ER	estrogen receptor
ERE	estrogen response element
fCSA	fiber cross-sectional area
FOXO	forkhead box O
GPCR	G-protein-coupled receptor
GSK3β	glycogen synthase kinase 3β
IGF-1	insulin-like growth factor
IGF-1R	insulin-like growth factor receptor
IGFBP	insulin-like growth factor binding protein
IRS1	insulin receptor substrate-1
LABC	levator ani/bulbocavernosus
MAFbx	muscle atrophy F-box or atrogin-1
MGF	mechano growth factor
MHC	myosin heavy chain
mTOR	mammalian target of rapamycin
MuRF1	muscle ring finger-1
NT-3	neurotrophin-3
ORX	orchiectomy
PGC-1α	peroxisome proliferator-activated receptor gamma co-activator-1α
PGC-1β	peroxisome proliferator-activated receptor gamma co-activator-1β
PI3K	phosphatidyl inositol-3 kinase
SCI	spinal cord injury
SHBG	sex-hormone binding globulin
SNB	spinal nucleus of the bulbocavernosus
T	testosterone
TCF-4	T-cell factor-4
TE	testosterone enanthate
TrkB	tyrosine receptor kinase B
TrkC	tyrosine receptor kinase C
TRT	testosterone replacement therapy
